# Knowledge, attitudes and practices survey of cardiac rehabilitation among cardiologists and cardiac surgeons in Lebanon

**DOI:** 10.1186/s43044-021-00212-2

**Published:** 2021-10-14

**Authors:** Rebecca Farah, Wim Groot, Milena Pavlova

**Affiliations:** 1 Department of Health Services Research, CAPHRI, Faculty of Health, Medicine and Life Sciences, Maastricht University Medical Center; Maastricht University, P.O. Box 616, 6200 MD Maastricht, The Netherlands; 2 Department of Rehabilitation and Physical Therapy (Group A); Delta-Chirec Hospitals Group, Brussels, Belgium

**Keywords:** Cardiac rehabilitation, Prevention, Exercises, Lifestyle, Barriers, Lebanon

## Abstract

**Background:**

Cardiovascular diseases (CVDs) are among the leading causes of morbidity and mortality worldwide. Over three quarters of the cardiovascular deaths take place in low and middle-income countries. Despite the benefits, Cardiac Rehabilitation (CR) is still not routinely and not universally available. Numerous studies have found that barriers to access to CR are correlated with providers, patients and environment characteristics. This first national survey on CR in Lebanon assesses the knowledge, attitudes and practices among physicians. In addition, the study identifies what the main barriers to access to CR are and provides suggestions for the implementation of CR in the country.

**Results:**

The response rate was 41.5% (*n* = 83). Results show that the cardiologists have medium level of knowledge about CR and its multidisciplinary content. Physicians support the implementation of a comprehensive CR program in the country. 50% of the physicians recommended first to solve the financial issues before implementing a CR program. Supplementary learning about the benefits of CR is highly recommended to enroll more patients with CVD into CR. In addition, the lack of specialists in the field, lack of motivation for patients to enroll to CR and inconvenient location of the rehabilitation centers were identified as major barriers by the respondents.

**Conclusions:**

The role of physicians in promoting patient enrolment should be optimized and exploited in the country. The access barriers identified can help to develop CR programs and to improve CR referral and enrolment rates. Funds from private parties and a budget from the government are needed to launch new CR programs in the country. Further research is needed to provide evidence on the CR benefits in Lebanon and to motivate policy-makers to place priority on the establishment of a comprehensive CR program in the country.

**Supplementary Information:**

The online version contains supplementary material available at 10.1186/s43044-021-00212-2.

## Background

Cardiovascular diseases (CVDs) are the number one cause of death globally: more people die annually from CVDs than from any other cause. An estimated 17.9 million people died from CVDs in 2016, representing 31% of all global deaths. Of these deaths, 85% are due to heart attack and stroke, according to the World Health Organization report [1]^.^ Over three quarters of the CVDs deaths take place in low- and middle-income countries [2, 3].

Reducing risk factors such as tobacco use (including water-pipe smoking), an unhealthy diet, obesity, and physical inactivity are the starting point to decrease the burden of CVDs in low-, middle- and high-income countries [4, 5]. In addition, there is evidence of a positive effect of Cardiac Rehabilitation (CR) for CVDs patients [6]. CR can help to achieve a reduction in premature mortality from CVDs. A WHO report on CR in low- and middle-income countries suggests that all patients with CVDs should have access to CR, and healthcare providers and patients and their families should be aware of CR [7].

CR is defined as “*a multifactorial and comprehensive non-pharmacological intervention in secondary prevention, designed to limit the [patho]physiologic and psychological effects of cardiovascular disease. CR includes components of diet, health education, advice on cardiovascular risk reduction, physical activity and stress management*” [8]. CR, as a medically supervised exercise training, has the potential to act as a catalyst for promoting other facets of rehabilitation, including risk factor modification through therapeutic lifestyle changes and optimization of psychosocial support [9–14].

Evidence on the benefits of CR suggests that CR may reduce all causes of mortality by as much as 28% and may reduce cardiac mortality by up to 31% [15–20]. Also, reduced morbidity and unplanned hospital admissions in addition to improvements in exercise capacity, quality of life and psychological well-being are well-recognized CR befits reported in international guidelines [20–23].

Despite the evidence on the benefits and clinical recommendations from the American College of Cardiology and American Heart Association, CR is still not universally available in low- and middle-income countries [24, 25]. CR is also not broadly adopted in the daily practice of physicians in high-income countries as a complementary and essential non-pharmacological intervention in the case of CVDs due to multifactorial causes [26]. Overall, CR is still frequently neglected and forgotten as part of the treatment of CVDs patients [27].

This study focuses on CR in Lebanon. Appendix 1 shows the rate of CVDs in Lebanon [2]. CVDs represent half of all deaths in Lebanon [2]. Therefore, controlling for cardiovascular risk factors is essential to improve the health of the population and to reduce the burden of CVDs. At the time of this study, in 2018, no comprehensive CR program had been implemented in Lebanon. Awareness campaigns have been implemented by the Lebanese Ministry of Public Health on CVDs and unhealthy behavior of the Lebanese population, such as a lack of physical activity, obesity, high level of smoking (for example, Lebanon ranks first in water-pipe use in the world) and a high level of CVDs deaths. Nevertheless, cardiovascular preventive programs such as CR are not a priority in Lebanon.

The objective of this study is to identify the knowledge, attitudes and practices of Lebanese cardiologists about CR. In particular, this study assesses the discharge treatment recommendations for patients within CVDs in Lebanon. Key barriers faced by cardiologists and cardio-surgeons to refer patients to CR are also identified. This study is descriptive and is based on the Knowledge, Attitude and Practice (KAP) framework. The study also provides suggestions for the future development of CR and takes into consideration the recommendations of cardiologists and cardiac surgeons for further expansion of CR practice.

## Methods

A quantitative cross-sectional design was adopted using a structured, self-administrated KAP questionnaire. The survey was conducted during the 13^th^ International conference of the Lebanese Cardiology Society (LCS) & the 4th Middle East Heart Failure Meeting held in December 2018, in Beirut, Lebanon. The participants were cardiologists and cardiac surgeons in Lebanon. Ethical approval to conduct this survey was obtained from the President of the LCS, and from the ethical committee of the society.

All medical doctors who attended the conference were invited to fill in the questionnaire. These included both Lebanese and foreign medical doctors. No selection of respondents was made. All conference participants could take the survey. The participants did not receive any incentive to take part in the study. The questionnaire was distributed by hand by the LCS board and the filled-in questionnaires were collected by the organizers of the 3-day LCS conference. In total, 83 out of 200 conference participants returned a filled-in questionnaire.

The self-administrated questionnaire was designed based on a literature review. This questionnaire was validated by experts in the field, such as cardiologists from the LCS. The questionnaire was updated according to their suggestions. The questionnaire was based on the three components of the KAP framework of the WHO (27) and contained 25 questions. We added two components: one about barriers faced by cardiologists and one about the socio-demographic data of the respondents. Appendix 2 shows the 25 questions asked to the physicians.

The questionnaire was divided into 5 parts:

- Part 1 covered questions on knowledge of physicians about CR
- Part 2 explored the attitudes of physicians on CR
- Part 3 assessed the daily practices of physicians on CR
- Part 4 investigated the barriers faced by physicians when referrer patients to CR
- Part 5 studied the socio-demographic data of the responders.

The questionnaire was anonymous. Written informed consent was obtained from each participant at the beginning of the questionnaire. The wording of questions was pre-tested before the survey. The English version of the questionnaire was used in the survey.

After the data collection and screening, data entry, cleaning and analysis were done by using the software package SPSS version 24. The analysis consisted of descriptive statistics, namely frequencies, mean, median, standard deviation, percentage for each response variable.

## Results

In total, 83 questionnaires were gathered among the 200 participants in the conference. Thus, the overall response rate was 41.5%. Results are shown in the tables below.

### Socio-demographic characteristics

Table 1 shows that 57.7% of the physicians who participated in the study were cardiologists. More than half of the participants (62.3%) had graduated in Lebanon. Respondents were for 76.6% males and 23.4% were females. In total, 29.9% of the participants were 40–50 years old and 28.6% were younger than 35. In addition, more than half of the participants (54.5%) had 10 to 15 years of practice experience. In total, 60.3% of the participants practiced in urban areas and 66.2% practiced in a hospital.

**Table 1:** Socio–demographic characteristics of the respondents

Age	< 35	N (%)	22 (28.6%)	Mean	2.4
	35–40	N (%)	19 (24.7%)	Median	2
	40–55	N (%)	23 (29.9%)	SD	1.17
	55–65	N (%)	9 (11.7%)		
	> 65	N (%)	4 (5.2%)		
		N total	77		
Gender	Male	N (%)	59 (76.6%)	Mean	1.8
	Female	N (%)	18 (23.4%)	Median	2
		N total	77	SD	0.487
Function	Cardiologist	N (%)	45 (57.7%)	Mean	2.17
	Cardiac surgeon	N (%)	8 (10.3%)	Median	1
	Manager	N (%)	4 (5.1%)	SD	1.6
	Cardiology fellow	N (%)	9 (11.5%)		
	MD	N (%)	11 (14.1%)		
	Others	N (%)	1 (1.3%)		
		N total	78		
Education	Lebanon	N (%)	33 (53.2%)	Mean	1.85
	US	N (%)	5 (8.1%)	Median	1
	EU	N (%)	19 (30.6%)	SD	1.16
	Others	N (%)	5 (8.1%)		
		N total	62		
Work Location	Rural	N (%)	27 (37%)	Mean	1.65
	Urban	N (%)	44 (60.3%)	Median	2
	Both	N (%)	2 (2.7%)	SD	0.532
		N total	73		
Place of graduation	Lebanon	N (%)	48 (62.3%)	Mean	1.85
	US	N (%)	2 (2.6%)	Median	1
	EU	N (%)	17 (22.1%)	SD	1.16
	Others	N (%)	10 (13%)		
		N total	77		
Work location office	Hospital settings	N (%)	49 (66.2%)	Mean	1.59
	Private office	N (%)	6 (8.1%)	Median	1
	Both	N (%)	19 (25.7%)	SD	0.87
		N total	74		
Number of Years of practice	< 5	N (%)	22 (28.6%)	Mean	2.59
	10-May	N (%)	12 (15.6%)	Median	3
	15-Oct	N (%)	42 (54.5%)	SD	1.22
	> 15	N (%	1 (1.3%)		
		N total	77		

### Knowledge about CR

Table 2 presents the results on the level of knowledge about CR. The table shows that almost one-third (31.3%, *n* = 26) of the participants stated that they had good knowledge of CR and 4.8% (*n* = 4) said they had very poor knowledge of CR. Regarding the content of the program, 31.3% of participants (*n* = 26) stated they had a medium level of knowledge of the multidisciplinary components of CR and 10.8% (*n* = 9) had very poor knowledge of the multidisciplinary CR components.

**Table 2:** Knowledge about cardiac rehabilitation (CR) among the respondents

Knowledge about CR—level	Very poor	N (%)	4 (4.8%)	Mean	3.28
	Poor	N (%)	14 (16.9%)	Median	3
	Medium	N (%)	29 (39.9%)	SD	1.04
	Good	N (%)	26 (31.3%)		
	Excellent	N (%)	10 (12%)		
		N Total	83		
Knowledge about CR—content	Very poor	N (%)	9 (10.8%)	Mean	2.93
	Poor	N (%)	22 (26.5%)	Median	3
	Medium	N (%)	26 (31.3%)	SD	1.16
	Good	N (%)	17 (20.5%)		
	Excellent	N (%)	9 (10.8%)		
		N Total	83		

### Attitude towards CR

Table 3 depicts the results on attitudes towards CR. More than half of the physicians in our study (55.4%, *n* = 46) agreed that a patient who is stable after Acute Coronary Syndrome could be enrolled into CR. Half of the participants (51.8%, *n* = 43) thought that CR in Lebanon could be effective. Less than half of responders (53.2%, *n* = 33) considered that access to an outpatient CR center could have an added value. Overall, 52.4% (*n* = 43) of the physicians in our sample strongly agreed that quality of the treatment would increase for patients who have received CR. Table 3: Attitude of cardiologists towards CR (*n* = 83).

**Table 3:** Attitude toward cardiac rehabilitation (CR) among the respondents

Do you think that a patient is stable post ACS could be enrolled into a CR? Please rate:	Strongly agree	N (%)	23 (27.7%)	Mean	1.95
	Agree	N (%)	46 (55.4%)	Median	2
	Neutral	N (%)	10 (12.0%)	SD	0.81
	Disagree	N (%)	3 (3.6%)		
	Strongly disagree	N (%)	1 (1.2%)		
		N Total	83		
Do you think that CR in Lebanon could be effective?	Strongly agree	N (%)	24 (28.9%)	Mean	1.927
	Agree	N (%)	43 (51.8%)	Median	2
	Neutral	N (%)	14 (16.9%)	SD	0.745
	Disagree	N (%)	2 (2.4%)		
	Strongly disagree	N (%)	0		
		N Total	83		
Do you consider that access for an outpatient CR center is an added value in the country?	Strongly agree	N (%)	34 (41%)	Mean	1.734
	Agree	N (%)	38 (45.8%)	Median	2
	Neutral	N (%)	10 (12.0%)	SD	0.717
	Disagree	N (%)	1 (1.2%)		
	Strongly disagree	N (%)	0		
		N Total	83		
Do you think that the outcome will be improved if your patients are enrolled in a CR	Strongly agree	N (%)	43 (52.4%)	Mean	1.6
	Agree	N (%)	28 (34.1%)	Median	1
	Neutral	N (%)	11 (13.4%)	SD	0.715
	Disagree	N (%)	0		
	Strongly disagree	N (%)	0		
		N Total	82		

### Practice of CR referrals

Table 4 presents the results on the practice regarding CR. The table shows that more than half of the physicians in our study (53%) would refer patients to CR after discharge from the hospital. Results show that 36.1% (*n* = 30) of the cardiologists consider “Heart Failure (HF) patients” as the main type of patient to be referred to CR. The second type of patients to be referred for CR are post valve surgery patients (26% *n* = 22). More than half of the participants in the study, 65.1% (*n* = 54), stated that it would be difficult to refer patients to CR in Lebanon. In total, 73.8% (*n* = 59) of the participants think that action must be taken by insurance companies, professional health givers, physicians, policy providers and the Ministry of Health to introduce CR programs in the country. Only 3.9% (*n* = 3) stated that they would never refer a patient to a CR program if such program would become available in the future and 48.7% (37) indicated that they already sent patients to CR 3–5 times per month. After discharging the patient from hospital, 48.1% (*n* = 38) of physicians in our study ask their patients to start a rehabilitation program. In total, 35.4% of the respondents request their patients to exercise a bit and 1.3% recommend to their patients to get bedrest. Table 4: Practice of cardiologist regarding CR (*N* = 83).

**Table 4:** Practice of cardiac rehabilitation (CR) by the respondents

When would you refer a patient to start a CR?	Starting in the hospital settings	N (%)	23 (27.7%)	Mean	1.92
	Directly after the hospital discharge			Median	2
	4 weeks or more after the discharge	N (%)	44 (53.0%)	SD	0.711
	Would not refer				
		N (%)	15 (18.1%)		
		N (%)	1 (1.2%)		
		N Total	83		
What kind of patients do you consider suitable for CR after discharging from hospital?	HF/CAD/Angina/ACS	N (%)	30 (36.1%)	Mean	3.5
	CABG	N (%)	10 (12%)	Median	3
	Post valve surgery	N (%)	2 (2.4%)	SD	2.43
	Pacemaker	N (%)	22 (26.5%)		
	Post vascular surgery	N (%)	1 (1.2)		
	All of the above	N (%)	18 (21.7%)		
		N Total	83		
Would it be difficult to refer patients to CR in the country?	Yes	N (%)	54 (65.1%)	MEAN	1.4
	No	N (%)	29 (34.9%)	Median	1
		N Total	83	SD	0.644
Who should take initiative to initiate CR in the country?	Insurance companies	N (%)	5 (6.3%)	Mean	4.31
	Professional care givers	N (%)	6 (7.5%)	Median	5
	Physicians	N (%)	7 (8.8%)	SD	1.26
	Policy makers	N (%)	3 (3.8%)		
	Ministry of Health	N (%)	59 (73.8%)		
		N Total	80		
How frequently would you send patients to CR if CR would have started?	3–5 times per month	N (%)	37 (48.7%)	Mean	1.85
	3–5 times per week	N (%)	21 (27.6%)	Median	2
	1–2 times per month	N (%)	13 (17.1%)	SD	1.05
	Once every 6 months	N (%)	2 (2.6%)		
	Never	N (%)	3 (3.9%)		
		N Total	76		
After discharge physicians asked patients to:	To do nothing, to be at rest	N (%)	1 (1.3%)	Mean	3.59
	To exercise a bit	N (%)	28 (35.4%)	Median	3
	To go to fitness club	N (%)	11 (13.9%)	SD	1.41
	To take it easy	N (%)	1 (1.3%)		
	To start a rehab program	N (%)	38 (48.1%)		
		N Total	79		

### Barriers related to CR

In Table 5, the results on barriers faced by physicians are presented. It shows that nearly all participants in our study (77.7%, *n* = 59) face barriers to refer patients to CR. Results presented in Table 5 demonstrate the kind of barriers that physicians face. The major barriers are a lack of specialists in the field, followed by a lack of motivation for the patient to enroll in CR and the location of the center.

**Table 5:** Barriers to access cardiac rehabilitation (CR) according to the respondents

Existence of barriers to refer patients to the CR	Yes	N (%)	59 (77.6%)	Mean	1.34
	No	N (%)	17 (22.4%)	Median	1
		N Total	76	SD	1.19
Kind of barriers faced by physicians	Lack of specialists	N (%)	18 (23.7%)	Mean	5.14
	Lack of knowledge	N (%)	3 (3.9%)	Median	7
	Lack of motivation	N (%)	4 (5.3%)	SD	2.54
	Cost of care	N (%)	1 (1.3%)		
	Localization of the center	N (%)	4 (5.3%)		
	All of the above	N (%)	46 (60.5%)		
		N Total	76		

### Support for CR

Results shown in Table 6 indicate that 84.6% (*n* = 66) of the participants in our study will fully support an inpatient CR program, 96.2% (*n* = 75) will support a future outpatient CR and 85.9% (*n* = 67) will support home-based cardiac tele-rehabilitation. Table 6: Support from Physicians to all kinds of CR (*N* = 83).

**Table 6:** Support for Cardiac Rehabilitation (CR) among to the respondents

Support for inpatient CR	Yes	N (%)	66 (84.6%)	Mean	1.15
	No	N (%)	12 (15.4%)	Median	1
		N Total	78	SD	0.363
Support for outpatient CR	Yes	N (%)	75 (96.2%)	Mean	1.03
	No	N (%)	3 (3.8%)	Median	1
		N Total	78	SD	0.19
Support for home-based cardiac tele- rehabilitation	Yes	N (%)	67 (85.9%)	Mean	1.16
	No	N (%)	11 (14.1%)	Median	1
		N Total	78	SD	0.467

### Respondents’ recommendations for CR

Half of the respondents (50%) recommended that first the financial issues need to be solved before implementing a CR program in Lebanon, and second that CR needs to be covered by insurance and government policies. The third recommendation by more than a quarter of the participants was related to additional education for medical doctors and public awareness about CR.

## Discussion

This study is the first to assess CR in Lebanon. Our findings show that physicians have some knowledge about CR and its components despite the absence of comprehensive CR programs in the country. The majority of the respondents strongly approved of CR and recognized its added value, as well as the beneficial CR outcomes. Regarding their practice, nearly all participants responded that it is difficult to refer a patient to CR. They expect to face barriers if a comprehensive CR program is implemented in the near future in Lebanon. Therefore, it was relevant to identify what kind of barriers respondents considered relevant in the case of CR, as well as what their suggestions and recommendation were for the further development of CR programs.

Our results found a medium level of knowledge of providers about CR. Several studies conducted in other countries on this topic, have found a correlation between knowledge and health education. For example, one study found that a lack of knowledge on CR is due to a deficiency of health education among both inpatient and outpatient providers, specifically on the importance of CR in improving outcomes in cardiac patients [36, 37]. The main barriers to CR referrals found in our study, according to the respondents, were the lack of specialists in the country, followed by a lack of motivation of the patient to enroll in a program and the location of the center, which is also an important factor. As suggested in the literature [37], despite the evidence on CR, the rate of patients’ enrollment in CR is very low, and even in high-income countries, cardiologists referred only 20 to 30% of their patients.

According to one study conducted on global access to CR, worldwide, there is low availability of CR; only 38.8% of countries globally have CR programs. Specifically, 68.0% of high-income and 23% of low- and middle-income countries (28.2% for middle- and 8.3% for low-income countries) have CR. CR density estimates ranged from 1 program per 0.1–6.4 million inhabitants [27]. In the USA, for example, participation rates range from 14 to 55% after myocardial infarction; in the UK, 28,6% of eligible patients were enrolled in CR; in Canada, about 30% of cardiac inpatients enroll in CR [6]. The reasons why cardiac patients are not participating in CR programs, are multifactorial and are related to a non-powerful enrollment after the referrals, no motivation of the patients, lack of insurance coverage of CR and inconvenient location of the CR center. A study conducted in Canada found that more knowledge and familiarity with CR guidelines among healthcare providers, is associated with higher self-reported referral rates [28].

Moreover, our findings showed that one of the key barriers to CR referrals was a lack of motivation of the patients to adhere to a program, as stated by respondents in our study. Several studies on providers barriers to refer patients to CR [36] have demonstrated that physicians’ attitude and behavior are key factors affecting patient’s adherence to the program. According to one systematic review, the strength of the primary care physician recommendations is highly correlated with patients’ adherence to CR [29]. One study published in the Journal of the American College of Cardiology in 2009, found that the strongest determinant of patient participation in CR is the strength of the recommendation of the physician making the referral. If the physician believes and conveys the importance “passionately”, patients are more likely to participate [30].

One study on physician attitudes toward CR in the Philippines has shown that lower CR enrollment is correlated with cardiologists who are more uncertain about the benefits of a CR program [29]. As reported in a systematic literature review, a large study conducted on 22 666 cardiac inpatients eligible for CR, found that patients who enrolled in a program received strong physician support to start CR, whereas those who did not enroll received either a weak or no recommendation at all [31]. Other studies found that medical specialties play an important role in the referral to CR [30]. Generalists were less likely to referrer patients to CR compared with specialists, such as cardiologists or cardiac surgeons at the discharge time. Additionally, the literature suggests that it will be better for patients to be seen by a specialist during their hospitalization and to be referred to CR prior to their hospital discharge to increase the level of participation in a CR program [32, 33]. The procedure is important for the enrollment of the patient to CR. A study reported referral rates of 48% among patients with percutaneous coronary intervention and 91% of patients who underwent cardiac surgery. The studies found that the strongest predictor of CR referral was the hospital performing the procedure [34].

Our study has also explored the recommendations of participants for the further development of CR in Lebanon. Results showed that there is a need to solve the financial issue of covering patients who enroll in CR and a need to change the Lebanese healthcare system for this. The healthcare system in Lebanon is funded more through private than public resources, and it has been overstretched by the increase in refugees from neighboring countries. No national strategic plan is available for the implementation of CR centers, and no budget is given from the government for CR. Our results demonstrate that physicians support all forms of CR, including inpatient, outpatient and home-based programs. Likewise, respondents in our study suggested more health education for providers and public awareness of CR benefits.

After the survey was conducted in 2019, two CR programs have opened in two hospitals in Lebanon. One in Beirut West and another one in Mount-Lebanon area, which will facilitate the access for patients suffering from CVD in the Beirut district. As suggested, barriers correlated to physicians’ attitude and practice need to be overcome by promoting the benefits of the programs among physicians in order to have more powerful and enthusiastic endorsement after discharge of the patients. Also, another study suggested that a “motivational letter” provided to the patients can be helpful to remind the physicians to initiate a CR referral 35. To have a strong endorsement in every medical establishment, CR must be in the procedure of the treatment for every CVD and must be cover by insurance and government.

## Limitations

Our study has some limitations. The survey was only done in English and not in Arabic or French. Although we recognize this limitation, we do not expect that it had a substantial influence on the results because the participants were professionals in the field of the study able to communicate in English. Nevertheless, some of the questions might have seemed hypothetical to the respondents since CR is largely absent in Lebanon. It is unclear how actual experience with referrals to CR in Lebanon would have influenced their responses. Another limitation was the difficulty to properly read the suggestions and comments of three respondents due to unclear handwriting. Also, we cannot exclude self-selection bias. Specifically, participants in the survey might have been more interested in CR and more positive about CR than the average medical doctor. This might have skewed the results toward positive opinions. The participants were a heterogeneous group, e.g. only about half of them were actually practicing in Lebanon and about two thirds were practicing CT surgeons or cardiologists. Moreover, the response rate is not very high, which limits the possibility to extrapolate our findings to the entire study population.

## Conclusions

As indicated by the study results and their discussion, Lebanese healthcare providers need more information and training on CR, which suggests that such education programs are important to develop. Supplementary learning about the benefits of CR is highly recommended to enroll more patients with CVD into CR. It is important to integrate CR in the procedure of CVD management in every hospital in Lebanon. There is also a need to provide coverage of CR in Lebanon. A significant endorsement of CR by a specialist at the hospital discharge, with a convincing explanation of CR benefits to the patients, is crucial to have a higher enrollment of patients to the new CR programs. By ensuring that CR is available to heart patients and expenses are covered, a reduction in cardiac morbidity and mortality can be expected at a national level, which is the top chronic disease problem in the country. Finally, the financial barrier needs to be overcome with a call for funding and further researches to provide evidence for policy.

## Key message of this study

### What is already known on this subject?

- Benefits of CR, providers barriers to enroll in a CR

### What might this study add?

- First study assessing CR in Lebanon among physicians
- Suggestions for implementation of CR in the country
- Local barriers identified and how to overcome
- Need of solving the financial issue to cover CR
- Need to have a policy from the government

### How might this impact on clinical practice?

- Need of a good communication between physicians and patients
- Timing of the communication and by a specialist must be consider
- CR need to be routinely prescribed, be inside the procedure of the management of the CVD
- Importance to enroll in a CR for burden in CVD in 2030
- To change bad habits of patients
- Suggestion for CR to reduce CVD death in Lebanon

## Supplementary Information

**Additional file 1**. Timing of communication by a specialist needs to be considered.

## Data Availability

Data are stored in Maastricht University and are available upon request if needed.

## Abbreviations

CVDs: Cardiovascular diseases
CR: Cardiac Rehabilitation
WHO: World Health Organization
SPSS: Statistical Package of Social Sciences
KAP: Knowledge Attitude and Practice
LCS: Lebanese Cardiology Society
