# Supplementary material for: Knowledge, attitudes and practices survey of cardiac rehabilitation among cardiologists and cardiac surgeons in Lebanon
Source: Egypt Heart J. 2021 Oct 14;73:87. doi: 10.1186/s43044-021-00212-2 (PMC8517050; doi:10.1186/s43044-021-00212-2)
Supplement: Supplementary file 1 — Additional file 1. Timing of communication by a specialist needs to be considered. [file 43044_2021_212_MOESM1_ESM.docx]

Appendix

Appendix 1: CVDs in Lebanon (WHO source)

Appendix 2: Survey questionnaire
